# Refining Expert Recommendations for Implementing Change (ERIC) strategy surveys using cognitive interviews with frontline providers

**DOI:** 10.1186/s43058-023-00409-3

**Published:** 2023-04-21

**Authors:** Vera Yakovchenko, Matthew J. Chinman, Carolyn Lamorte, Byron J. Powell, Thomas J. Waltz, Monica Merante, Sandra Gibson, Brittney Neely, Timothy R. Morgan, Shari S. Rogal

**Affiliations:** 1 Center for Health Equity Research and Promotion, VA Pittsburgh Healthcare System, Building 30, Room 2A113, University Drive C (151C), Pittsburgh, PA 15240-1001 USA; 2 RAND Corporation, Pittsburgh, PA USA; 3 Center for Mental Health Services Research, Brown School, Washington University in St. Louis, St. Louis, MO USA; 4 Center for Dissemination & Implementation, Institute for Public Health, Washington University in St. Louis, St. Louis, MO USA; 5 Division of Infectious Diseases, John T. Milliken Department of Medicine, School of Medicine, Washington University in St. Louis, St. Louis, MO USA; 6 Department of Psychology, Eastern Michigan University, Ypsilanti, MI USA; 7 Division of Gastroenterology, Hepatology, and Nutrition, University of Pittsburgh, Pittsburgh, PA USA; 8 Gastroenterology Section, VA Long Beach Healthcare System, Long Beach, CA USA; 9 Department of Medicine, University of California, Irvine, CA USA; 10 Department of Surgery, University of Pittsburgh, Pittsburgh, PA USA

**Keywords:** Implementation strategies, Expert Recommendations for Implementing Change, Practitioners, Implementation practice, Cognitive interviews

## Abstract

**Background:**

The Expert Recommendations for Implementing Change (ERIC) compilation includes 73 defined implementation strategies clustered into nine content areas. This taxonomy has been used to track implementation strategies over time using surveys. This study aimed to improve the ERIC survey using cognitive interviews with non-implementation scientist clinicians.

**Methods:**

Starting in 2015, we developed and fielded annual ERIC surveys to evaluate liver care in the Veterans Health Administration (VA). We invited providers who had completed at least three surveys to participate in cognitive interviews (October 2020 to October 2021). Before the interviews, participants reviewed the complete 73-item ERIC survey and marked which strategies were unclear due to wording, conceptual confusion, or overlap with other strategies. They then engaged in semi-structured cognitive interviews to describe the experience of completing the survey and elaborate on which strategies required further clarification.

**Results:**

Twelve VA providers completed surveys followed by cognitive interviews. The “Engage Consumer” and “Support Clinicians” clusters were rated most highly in terms of conceptual and wording clarity. In contrast, the “Financial” cluster had the most wording and conceptual confusion. The “Adapt and Tailor to Context” cluster strategies were considered to have the most redundancy. Providers outlined ways in which the strategies could be clearer in terms of wording (32%), conceptual clarity (51%), and clarifying the distinction between strategies (51%).

**Conclusions:**

Cognitive interviews with ERIC survey participants allowed us to identify and address issues with strategy wording, combine conceptually indistinct strategies, and disaggregate multi-barreled strategies. Improvements made to the ERIC survey based on these findings will ultimately assist VA and other institutions in designing, evaluating, and replicating quality improvement efforts.

**Supplementary Information:**

The online version contains supplementary material available at 10.1186/s43058-023-00409-3.

Contributions to the literature

- To our knowledge, this is the first study evaluating how frontline health care providers understand the 73 implementation strategies within the Expert Recommendations for Implementing Change.
- In cognitive interviews, providers noted the areas to improve most strategy descriptions, with variability by strategy cluster.
- Providers described difficulty with implementation science jargon, strategy redundancies, vague operationalizations, and the unintended consequences of responding to strategy surveys.
- Our findings highlight the need to improve overall strategy clarity and develop project-tailored implementation glossaries to improve the accuracy of strategy reporting by lay providers.

## Background

Moving evidence-based practices (EBPs) into routine care settings to improve healthcare quality and outcomes requires the skillful selection of implementation strategies, defined as “methods or techniques used to enhance the adoption, implementation, and sustainability of a clinical program or practice” [1]. Still, it is estimated that it takes 17 years from the time of development for EBPs to achieve 50% penetration into routine clinical practice [2]. In addition to depriving people of the best care, these delays also mean that, by the time the evidence reaches people, it may already be out of date.

Implementation strategies can vary widely, as can their labels, definitions, and applications. Since meaning often derives from naming, it is semantically important to accurately describe strategies, understand their referents, and their relationships to other strategies and contexts. It is likewise important that each strategy be clear and distinct from all other strategies as to understand which strategies and combinations of strategies enhance EBP adoption, implementation, and sustainment. However, implementers rarely justify the selection of certain strategies over others [3], despite Proctor et al.’s [1] guidance from 2013 to thoughtfully select and specify strategies. Failing to characterize strategies appropriately has hampered advancement of the science of implementation and its practical applications.

To generate a common nomenclature for implementation strategies and facilitate standardization of research methods and replication, the Expert Recommendations for Implementing Change (ERIC) study engaged experts in modified Delphi approach and concept mapping to 1) refine a compilation of implementation strategies and 2) develop conceptually distinct categories of implementation strategies [4]. This led to a compilation of 73 discrete implementation strategies, which were further organized into nine thematic clusters, including financial, infrastructure, supporting clinicians, education, and patient-facing strategies, among others [5].

As part of a Veterans Health Administration (VA) program evaluation of a national hepatitis C virus (HCV) quality improvement (QI) initiative in 2015, we developed a novel survey of ERIC implementation strategies to longitudinally identify strategies used throughout the course of an initiative [6]. We have previously described our ERIC strategy survey development process [7]. In brief, the ERIC surveys present 73 strategies by cluster and offer a binary choice of having done or not done a strategy in the past year. Parenthetical examples are tailored to the EBP of interest (e.g., hepatitis C treatment, advanced liver disease, HIV prevention and care, opioid safety measures). Five years of longitudinal data with hundreds of responses informed us about strategy use and effectiveness.

While these ERIC surveys have been employed to document strategy use across several clinical areas and prescribe strategies that may work, it is unclear how non-implementation scientists (specifically front-line health care providers) interpret survey items. Thus, the overarching goal of this study was to understand how non-implementation scientists interpreted and experienced the ERIC implementation strategy survey.

## Methods

### Design

This mixed-methods study was approved by the VA Pittsburgh Healthcare System Institutional Review Board (Pro00003422). Interview data were collected from providers focused on improving liver care across VA between October 2020 and October 2021. Participants were purposively selected based on their experience responding to multiple ERIC strategy surveys over the course of a national quality improvement initiative. Agreeable participants provided verbal consent, reviewed an online survey with the 73 strategy items, and participated in a cognitive interview about ERIC strategies. Cognitive interviewing, often used to learn about the perceptions of survey respondents, is a method in which individuals are invited to verbalize thoughts and feelings as they examine information—namely items on a survey [8]. Qualitative methods followed COREQ guidelines [9].

### Participants and data collection

We purposively selected participants who had completed a strategy survey at least three times within seven years in order to gauge the experience of those who repeatedly engaged with the survey over time. Two pilot interviews were conducted to review and refine the interview guide prior to starting the study. Thirty participants were invited to participate via email, 14 agreed, and 12 completed interviews. Participants completed a 15-min pre-interview survey in SurveyMonkey and a virtual interview via Microsoft Teams lasting 60–90 min. Semi-structured interviews included three parts and were guided by visual displays of the strategies in PowerPoint and PowerBI. Participants also provided their degree, role, and experience with quality improvement. All interviews were conducted by a master’s level qualitatively trained member of the study team (CL). Field notes were taken by two other team members (SG, BN). Interviews were digitally recorded and transcribed verbatim.

### Pre-interview survey development

The pre-interview survey paralleled the typical ERIC strategy survey and contained all 73 implementation strategies and asked the respondent to comment about their potential confusion and clarity with wording, or whether the strategy was similar or distinct from every other strategy. Participants were presented both the original generic ERIC strategies and the tailored ERIC strategies for the pre-interview survey. First, the pre-interview survey displayed the original generic ERIC strategies as to achieve the most consistent interpretation of strategies given participants had either responded to advanced liver disease and/or hepatitis C care surveys which had had uniquely tailored strategies.

### Interview development

As is typical in cognitive interviewing, the interview for this study was developed to accomplish three goals, including 1) understand users’ experience with survey completion, 2) evaluate issues with comprehension, and 3) identify multiple embedded and conceptually indistinct strategies, to determine which would be best combined versus disaggregated [8]. Typically, strategy interpretations were not “corrected” unless participants were highly confused (as reinforced by the interviewer through “there’s no right or wrong answer”). During interviews, both generic and strategies tailored to hepatitis C treatment were the reference points to draw out general perceptions and specific abstractions of strategy details.

Participants were asked semi-structured questions about their experiences with completing the survey. The interviewer followed a semi-structured script and the think aloud method [10] to ask questions about strategy comprehension. This included asking about strategy specifications based on Proctor et al.: “*For the strategies that you did report using, could you give further details on what your site did? If yes, what kinds of details (for example who did it, what did they do, who were the targets, when was it done/temporality, how often it was done/dose, outcomes addressed, and justification for doing)*?” [1].

Participants were asked to interpret a subset of strategies that were identified a priori by the study team as either (1) similar or potentially overlapping or (2) having multiple embedded strategies. The team reached consensus about ten strategy pairs that were potentially overlapping. For example, “work with educational institutions” and “develop academic partnerships” were considered overlapping strategies. Participants were asked to rate “*How clear is the difference to you?*” between the two strategies on a 4-item Likert scale (“very unclear,” “unclear,” “clear,” “very clear”), as well as to describe the difference between the strategies in their own words. In the instances when participants asked for more details on the strategy descriptions, the interviewer would refer to the complete original ERIC definition on the screen for the participant to read.

To define whether multiple strategies should be considered as an integrated process or two sequential activities, the study team independently read through the strategy survey and then discussed to consensus which strategies should be examined in detail through cognitive interviews. Ten strategies that had two or more components or multiple embedded strategies were divided into parts by their distinct verbs. For example, “capture and share local knowledge” was split into “capture local knowledge” and “share local knowledge,” and participants were asked to rate “*How often are these done together?*” on a 4-item Likert scale (“never,” “sometimes,” “usually,” “always”). The intent was to understand how often strategies with multiple embedded activities were done together, the timing of proposed multiple parts, and other relevant details.

### Data analysis

Analysis included several steps and was conducted in NVivo, Microsoft Excel, and Microsoft Word. First, pre-interview survey responses were summarized to evaluate the frequency of wording, concept, and similarity difficulties. Proportions in text exclude the two pilor interview participants. Second, two coders (CL, MM) used the rigorous and accelerated data reduction (RADaR) technique and content analysis to code and analyze interviews [11]. Rapid coding and analysis allowed us to identify data saturation. Coders used a priori codes based on the interview guides and generated new codes through a general inductive approach [12, 13]. A matrix template was used to organize and manage the data. Coding (CL, MM) was followed by discussion with a third coder (VY) for consensus. Then, all coders collectively identified the final themes.

## Results

### Participant characteristics

Twelve cognitive interviews were conducted with VA providers, who reported responding to an average of five of the seven surveys between 2015 and 2020. The 12 participants were geographically diverse and covered a range of areas of expertise (i.e., one MD, four PharmDs, five advanced practice providers, and two RNs). Half had quality improvement training in addition to clinical expertise, but none had prior implementation science or research training.

### Survey response process

#### Who should complete the survey

Most participants (83%) confirmed they were correctly identified as a key informant and felt comfortable reporting on implementation strategies. However, 33% also engaged other informants when responding to the survey. One participant explained, “What we typically do is I go through [the survey] individually, and then I review with our team…and then we made a general consensus” (P02). Nevertheless, participants qualified that response validity and reliability was contingent on how closely someone worked to the clinical effort in question.

#### How the survey should be introduced

Participants had several suggestions about introducing the survey to a clinical audience. They suggested that explaining how the data would be used would encourage responses. As one participant remarked, “That’s the only way it’s going to make them see how it matters to them” (P11).

#### The impacts of completing the survey multiple times

All participants completed the annual survey over multiple years, and half said their understanding of strategy questions increased over time. Most explained that, if they were unsure about a strategy’s meaning or use, that they would report *not* using that strategy (78%). One participant said, “[I] don’t even know what that really means, so I’m just gonna say no” (P08).

#### Comprehensiveness of the survey

When asked if there were any activities that were done but not included in the survey, participants did not suggest additional strategies. One participant said, “I don’t know how you would ever miss something” (P09).

### Language and wording

#### Using clinical language

Participants universally recommended minimizing implementation science jargon or “doublespeak” (P11). Many suggested adding more explicit parentheticals to highlight possible minor differences between strategies. This was particularly important for “when you’re dealing with clinical people…you may have to use less implementation science verbiage and sort of translate that into normal English that somebody is going to understand” (P10). One participant considered this tradeoff when adding strategy details: “It might make it longer but it, you might get more accurate responses” (P04). Participants expressed “brain fatigue” resulting from the current wording and length but that, with focus, they could understand the differences between strategies: “If I slow down and really think about it and kind of overanalyze it, because that’s what I tend to do, I think I can tell the difference” (P03). Several participants emphasized the need for the language to reflect the “real world” perspective. For instance, participant clinical background shaped interpretation of common words such as “visit,” “consultation,” and “technical assistance” in ways that may not have aligned with the intended ERIC definitions. One nurse asked, “What do you mean facilitate?” (P07).

### Asking about strategy “use” not “implementation”

Furthermore, many (67%) noted confusion about whether “implemented” referred to whether they *started* using a strategy or continued an ongoing strategy. As such, some respondents did not endorse strategies they were using, because they thought strategies that were ongoing but institutionalized and in the sustainment phase were not of interest.

### Organizing and specifying strategies

#### Clinicians’ ability to specify strategies

When asked whether they could specify Proctor et al. strategy details, participants confirmed they could feasibly and confidently provide information about the action, the frequency, and the justification for the strategy; however, they had more difficulty defining who performed and received the strategy, the outcomes that were targeted, and the stage of implementation. Those with more QI experience could better articulate strategy specifics, but everyone alluded to difficulty disaggregating how strategies were actually used in complex clinical environments. Interestingly, we did not observe differences in responses based on how many times a survey response was provided (three vs five vs seven) suggesting a possible plateau effect at three.

#### Challenges with variable strategy specification

Participants underscored that strategies operated at differing levels and had differing specificity in their descriptions. Participants noticed that certain strategies could be employed by a single provider, while others required a clinical team or leadership support. Regarding the timing and stage of strategy use, clinicians were readily able to distinguish pre-implementation and implementation timing but could not easily delineate which strategies were used for sustainment. Likewise, they noted several strategies had embedded dosing information (e.g., one-time vs ongoing education), while most did not specify dosing. As such, some strategies were perceived to be more nebulous or dynamic than other, more clearly delineated, standardized strategies.

### Placing less feasible strategies later in the survey

Participants were often frustrated with being asked about strategies that were perceived out of scope or out of their purview, suggesting “It leads to this sense of failure because you have not done something like work with an educational institution and then you start spinning in your brain like, “How would I even accomplish that?” (P11). Specifically, placing “Financial” cluster strategies at the beginning of the survey may have inadvertently discouraged participation because “we don’t have any control over that whatsoever” (P08).

#### Unintended uses of the survey

We observed several unintended consequences of participating in the survey. First, the survey served as a tool for ongoing tracking of activities and to anticipate responding to the strategy survey in the future years so “I didn’t have to rely on just my memory alone” (P03). Second, for a few participants, the survey was an “idea generator” and inspired future implementation: “each time we do the survey…you look at it as, ‘Oh, I have to do this’” (P02). Also, one participant recommended to ask “prospective questions…not what did you do in the past, but what do you plan on doing in the future?” (PL2).

### Strategy clarity

#### Strategies clarity varied

According to the participants with a pre-interview survey, most strategies (90%) had at least one confusing element for one or more respondents and half (48%) had at least two. Strategies were unclear due to wording (32%), conceptual confusion (51%), or similarity between strategies (51%) for one or more respondents. Table 1 presents the most confusing clusters and strategies as endorsed by participants. Strategies within the “Financial” cluster were the most unclear to this group of VA clinicians, both in terms of language and conceptually (mean total concerns 6.8, range among strategies 0 to 9). Conversely, clarity was highest for strategies in the “Engage patient” cluster (mean total concerns 0.8, range 0 to 2) and “Support Clinicians” (mean total concerns 1.2, range 0 to 3) clusters. Wording concerns were most likely in the "Provide interactive assistance" cluster, while conceptual concerns were most present in the "Change infrastructure" cluster. Strategies in the "Train and educate stakeholders" and “Adapt and tailor to context” clusters were perceived to have the most overlap between one another. Almost half of strategies (44%) had “Other” uncategorized confusion which primarily reflected perceptions of relevance to the VA setting such as with “Make billing easier”: “I wasn’t aware billing for patient care services could be altered at the local facility level? So, this question of the survey seemed odd” (P03).

**Table 1:** Survey user validity concerns

Original generic ERIC strategies	Tailored to hepatitis C ERIC strategies	Wording	Concept	Similarity	Other	Total
***Change infrastructure***						
11. Change physical structure and equipment	1. Change physical structure and equipment^a^	3	3	0	1	7
12. Change record systems	2. Change the record systems	2	5	1	1	9
13. Change service sites	3. Change the location of clinical service sites	0	0	0	0	0
62. Start a dissemination organization	4. Develop a separate organization or group responsible for disseminating HCV care	0	1	0	0	1
44. Mandate change	5. Mandate changes to HCV care	1	3	0	1	5
22. Create or change credentialing and/or licensure standards	6. Create or change credentialing and/or licensure standards^b^	1	4	0	1	6
10. Change liability laws	7. Participate in liability reform efforts that make clinicians more willing to deliver the clinical innovation	1	4	0	2	7
9. Change accreditation or membership requirements	8. Change accreditation or membership requirements^b^	1	5	1	1	8
***Financial strategies***						
1. Access new funding	9. Access new funding^a^	0	2	1	1	4
2. Alter incentive/allowance structures	10. Alter incentive/allowance structures	2	3	0	2	7
28. Develop disincentives	11. Provide financial disincentives for failure to implement or use the clinical innovations	0	0	0	4	4
34. Fund and contract for the clinical innovation	12. Respond to proposals to deliver HCV care	1	2	0	0	3
42. Make billing easier	13. Change billing	1	3	0	6	10
49. Place innovation on fee for service lists/formularies	14. Place HCV medications on the formulary	0	0	0	1	1
3. Alter patient/consumer fees	15. Alter patient fees	1	2	2	5	10
66. Use capitated payments	16. Use capitated payments	2	4	0	5	11
70. Use other payment schemes	17. Use other payment schemes	2	3	1	5	11
***Support clinicians***						
21. Create new clinical teams	18. Create new clinical teams	0	0	0	0	0
32. Facilitate relay of clinical data to providers	19. Facilitate the relay of clinical data to providers^b^	0	0	0	0	0
59. Revise professional roles	20. Revise professional roles	0	0	0	1	1
58. Remind clinicians	21. Develop reminder systems for clinicians	0	0	1	1	2
30. Develop resource sharing agreements	22. Develop resource sharing agreements	0	3	0	0	3
***Provide interactive assistance***						
33. Facilitation	s23. Use outside assistance often called “facilitation”	2	3	1	1	7
54. Provide local technical assistance	s24. Have someone from inside the clinic/center (“local technical assistance”) tasked with assisting the clinic^b^	0	2	2	0	4
53. Provide clinical supervision	25. Provide clinical supervision^b^	0	1	0	0	1
8. Centralize technical assistance	26. Use a centralized system to deliver facilitation	3	3	2	0	8
***Adapt and tailor to context***						
67. Use data experts	27. Use data experts to manage HCV data	0	0	2	0	2
68. Use data warehousing techniques	28. Use data warehousing techniques	2	2	2	0	6
63. Tailor strategies	29. Tailor strategies to deliver HCV care	0	2	1	0	3
51. Promote adaptability	30. Promote adaptability^b^	0	4	1	1	6
***Train and educate stakeholders***						
15. Conduct educational meetings	31. Conduct educational meetings^b^	0	0	2	0	2
16. Conduct educational outreach visits	32. Have an expert in HCV care meet with providers to educate them^b^	0	0	2	0	2
19. Conduct ongoing training	33. Provide ongoing HCV training^b^	0	0	2	0	2
20. Create a learning collaborative	34. Facilitate the formation of groups of providers and fostered a collaborative learning environment	0	0	2	0	2
29. Develop educational materials	35. Developed formal educational materials^b^	0	0	2	0	2
31. Distribute educational materials	36. Distribute educational materials	0	0	3	0	3
55. Provide ongoing consultation	37. Provide ongoing consultation with one or more HCV treatment experts	1	0	1	0	2
71. Use train-the-trainer strategies	38. Train designated clinicians to train others^b^	0	0	1	0	1
43. Make training dynamic	39. Vary the information delivery methods to cater to different learning styles when presenting new information	0	0	0	1	1
60. Shadow other experts	40. Give providers opportunities to shadow other experts in HCV	1	0	0	0	1
73. Work with educational institutions	41. Use educational institutions to train clinicians	3	1	2	0	6
***Develop stakeholder relationships***						
6. Build a coalition	42. Build a local coalition/team to address challenges	0	0	1	2	3
17. Conduct local consensus discussions	43. Conduct local consensus discussions^b^	2	3	0	1	6
47. Obtain formal commitments	44. Obtain formal written commitments from key partners that state what they will do to implement HCV care	0	0	0	2	2
57. Recruit, designate, and train for leadership	45. Recruit, designate, and/or train leaders	0	0	1	0	1
38. Inform local opinion leaders	46. Inform local opinion leaders about advances in HCV care	1	3	3	1	8
7. Capture and share local knowledge	47. Share the knowledge gained from quality improvement efforts with other sites outside your medical center^a^	0	0	0	1	1
35. Identify and prepare champions	48. Identify and prepare champions^a^	0	1	0	0	1
48. Organize clinician implementation team meetings	49. Organize support teams of clinicians, give them time to share the lessons learned and support one another’s learning^a^	0	1	2	0	3
64. Use advisory boards and workgroups	50. Use advisory boards and interdisciplinary workgroups to provide input into HCV policies and elicit recommendations	0	0	2	1	3
65. Use an implementation advisor	51. Seek the guidance of experts in implementation^a^	0	1	1	1	3
52. Promote network weaving	52. Build on existing high-quality working relationships and networks to promote information sharing and problem solving^b^	0	0	1	0	1
45. Model and simulate change	53. Use modeling or simulated change^b^	1	5	0	0	6
24. Develop academic partnerships	54. Partner with a university to share ideas	0	0	0	1	1
36. Identify early adopters	55. Make efforts to identify early adopters to learn from their experiences^b^	0	1	0	0	1
72. Visit other sites	56. Visit other sites outside your medical center to try to learn from their experiences	0	0	1	0	1
25. Develop an implementation glossary	57. Develop an implementation glossary	1	4	0	2	7
40. Involve executive boards	58. Involve executive boards	0	1	2	0	3
***Use evaluative and iterative strategies***						
4. Assess for readiness and identify barriers and facilitators	59. Assess for readiness and identify barriers and facilitators to change	0	0	0	1	1
18. Conduct local needs assessment	60. Conduct a local needs assessment^a^	0	0	0	0	0
23. Develop a formal implementation blueprint	61. Develop a formal implementation blueprint	0	2	0	1	3
61. Stage implementation scale up	62. Start with small pilot studies and then scale them up	0	0	1	0	1
5. Audit and provide feedback	63. Collect and summarize clinical performance data and give it to clinicians and administrators to implement changes in a cyclical fashion using small tests of change before making system-wide changes	0	0	3	0	3
14. Conduct cyclical small tests of change	64. Conduct small tests of change, measured outcomes, and then refine these tests^b^	0	0	3	0	3
26. Develop and implement tools for quality monitoring	65. Develop and use tools for quality monitoring	0	0	1	2	3
27. Develop and organize quality monitoring systems	66. Develop and organize systems that monitor clinical processes and/or outcomes for the purpose of quality assurance and improvement^a^^, b^	0	0	1	0	1
56. Purposely reexamine the implementation	67. Intentionally examine the efforts to promote HCV care^a,b^	1	2	2	0	5
46. Obtain and use patients/consumers and family feedback	68. Develop strategies to obtain and use patient and family feedback	0	0	0	0	0
***Engage consumers***						
41. involve patients/consumers and family members	69. Involve patients/consumers and family members^a,b^	0	1	0	0	1
50. Prepare patients/consumers to be active participants	70. Engage in efforts to prepare patients to be active participants in HCV care^b^	0	0	0	0	0
39. Intervene with patients/consumers to enhance uptake and adherence	71. Intervene with patients/consumers to promote uptake and adherence to HCV treatment^b^	0	0	0	0	0
69. Use mass media	72. Use mass media to reach large numbers of people^b^	0	1	0	1	2
37. Increase demand	73. Promote demand for HCV care among patients through any other means	0	1	0	0	1

Numbering refers to the order presented in the original ERIC study in the 1st column and the ERIC survey in the 2nd column

^a^Similar strategy

^b^Strategy with multiple embedded strategy

### Similar strategies

#### Some strategies should be combined

Of the 10 similar strategy pairs selected by the study team, participants suggested combining five, separating three, and were undecided on two (Table 2). Five of the 10 pairs were from the same ERIC cluster, while five were from different clusters; this did not impact whether strategies were perceived as similar or different. A nurse reflected many strategies were “synonyms of each other” and yet could not identify why a certain “word just sounds better” (P07). Beyond the pre-identified pairs of similar strategies, participants universally recommended to continue to “take out any redundancy” (PL2). Two of ten strategy pairs were difficult to discern, including “Facilitation” and “Provide ongoing consultation,” and “Conduct educational meetings” and “Conduct educational visits” because of the lack of detail in the labels and parenthetical examples.

**Table 2:** Similar strategies

Strategy 1	Strategy 2	Frequency of response: “very clear” or “clear” difference	Quote
***Keep strategies separate***			
Change accreditation or membership requirements	Create or change credentialing and/or licensure standards	67%	They’re totally different bodies that grant those credentials or license numbers, they’re very different. (P13)
Facilitate relay of clinical data to providers	Audit and provide feedback^a^	83%	The first box is talking about “How do we get…the information or data out to the providers?”…And then the second box seems like, “How do we evaluate how it was received or if it’s being implemented?” (P04)
Provide local technical assistance	Use an implementation advisor^a^	67%	So technical assistance is…if you have a dashboard, you have someone help you figure out how to find certain populations, how to navigate the dashboard. Whereas the implementation is more of, “Hey, this is what we have available for your site to help you with X, Y, and Z.”…So, implementation is strategies or tools versus the first one is helping you with how to use it. (P01)
***Strategies are so similar they can be combined***			
Develop and implement tools for quality monitoring	Develop and organize quality monitoring systems	25%	I mean, the word “organize” in the one is different. But I don’t know what the difference between “a quality monitoring system” and “a tool for quality monitoring” is. To me, that sounds like the same thing. (P12)
Tailor strategies	Promote adaptability	25%	So, when we’re tailoring strategies and promoting adaptability, when I go back and think about maybe examples, yeah it was all about how we can bring Hep C care to the patient and make that access point easier. So, I don’t think our team really distinguished between the two, and that was probably because I didn’t distinguish between the two. (P03)
Work with educational institutions	Develop academic partnerships^a^	33%	It sounds like on the left, it’s maybe a little less committal. You’re just sort of saying, “encouraging,” whereas on the right you’re “partnering.” (P06)
Involve patients and family members	Obtain and use patients and family feedback^a^	33%	That’s unclear as well. It just seems like it’s the same, whether you’re going to involve them or obtain and use their information. Either way, you’re still gonna involve the patient, consumers, and family to get the feedback, so it just seems like it’s a redundant question, maybe. (P10)
Intervene with patients to enhance uptake and adherence	Prepare patients to be active participants	33%	These seem to be similar, because you’re developing strategies for them to help you with their care for adherence and then preparing them. (P07)
***Undecided about separating or combining strategies***			
Facilitation	Provide ongoing consultation^a^	42%	When I think of “facilitation,” I think of a person in charge of some sort of process improvement project. When you use the word “consultation,” that has a clinical meaning, and so as a provider, you are talking about… a consultation [as a] patient specific review of clinical data with recommendations at the end. I don’t see that as being part of the clinical innovation. I see that as referring much more specifically to patient care activities. (P13)
Conduct educational meetings	Conduct educational visits	42%	Well, the first one, and if you could do that, that would be someone internally, “OK, we’re just going to do it.” And the other one would be an external trainer. (P05)

^a^Strategies are in different ERIC clusters

#### Including similar strategies can result in unintended overinterpretation

When asked for side-by-side comparisons, participants often overinterpreted the wording to distinguish strategies in ways that the ERIC group may not have intended. Others recognized there were “subtle differences” between strategies but also said “I can’t verbalize the difference very well” (P06). In a minority of instances, strategy “definitions made [the differences between strategies] more unclear” (P10). One advanced practice provider described the strategy target as the key to interpreting between strategies (e.g., was the strategy targeting clinicians as primary recipients or targeting clinicians to reach patients). The strategies “Facilitate relay of clinical data to providers” and “Audit and provide feedback” were clearly distinct “because you’re trying, you’re going to modify behavior in the second one. OK, ‘collect data’. You’re going to give it to them, and then you’re going to change what they do” (P05).

#### Patient-facing strategies often overlapped or were unclear

Participants interpreted “Intervene with patients to enhance uptake and adherence” to overlap with “prepare patients to be active participants” because of the patient-orientation, although no details of patient activities were described. Notably, as frontline providers, participants wondered about the lack of specificity in the patient-facing strategies. Some reinforced strategies “may seem duplicate to us, but you guys are obviously trying to get at two totally different things” (P08).

### Multiple embedded or multi-barreled strategies

Participants were asked to comment on their understanding of the composition of ten multi-barreled strategies (i.e., those with multiple embedded strategies). Furthermore, they were asked whether such strategies should remain as is or be divided into multiple strategies. Participants reported that all multi-barreled strategies included sequential steps in a process. Five strategies were considered to always or usually occur together, while five were considered less likely to co-occur together (Table 3). Overall, participants agreed “this is a chain of events that’s going to happen” (PL2), but they were more “hesitant on the timeline” (P07).

**Table 3:** Multiple embedded strategies

Strategy (part 1)	Strategy (part 2)	Frequency of response: strategy parts “always” or “sometimes” combined	Quote
***Assessed consensus: keep part 1 and part 2 combined***			
Develop quality monitoring systems	Organize quality monitoring systems	83%	Almost always, you need to put those two together to make sure we’re doing things correctly and have a way to measure. (P02)
Develop tools for quality monitoring	Implement tools for quality monitoring	75%	Developing and implementing quality monitoring takes a lot of time. We were really good at implementing things people had already developed for us; less probably effective at implementing or developing tools from the ground up. So, I think we did, usually, a very good job of keeping those ideas coupled. We did not develop a lot of our own tools ourselves. (P03)
Identify champions	Prepare champions	75%	I definitely think we would pick someone first and then get them ready. …[S]ometimes it could take a while too, depending on who you’re dealing with. (P05)
Obtain feedback	Use feedback	67%	I think that that would be done together as well, because once you obtain that feedback, then you see if you can utilize it and go forward with it. (P07)
Recruit and designate for leadership	Train for leadership	58%	It’s sequentially: you recruit or designate them and then you have to train them. (P12)
***Assessed consensus: do not combine part 1 and part 2***			
Assess for readiness	Identify barriers and facilitators	50%	I’m not sure that Part 1 is done very often. I think that strategy probably is more heavily the second part. (P13)
Fund for the clinical innovation	Contract for the clinical innovation	50%	Well within the VA, I’d say probably “Always,” because in order for clinical end of innovation to happen you have to have the funds in the contract to do it. (P07)
Capture local knowledge	Share local knowledge	42%	To capture local knowledge, you need to find out what your audience knows—this is how I interpret it. And then, Part Two, you need to share with others what the baseline knowledge is. (P14)
Change physical structure	Change equipment	33%	What they do is they kind of modify the clinic space and buy new stuff for the clinic space and that rarely happens–either of them. I don’t think either of those things happen very often. (P06)
Use advisory boards	Use workgroups	25%	Because sometimes you just need a workgroup and then sometimes you need to have advisory boards with it, so that’s a sometimes. Just depends on what you’re doing. (P07)

#### Some embedded strategies should remain as is

Participants recommended that five of the multi-barreled strategies remain together. These strategies focused on data and opinion leaders. For example, “Develop and organize quality monitoring systems” was seen as including two sequential but cohesive steps in one process (develop and then organize systems for monitoring quality). Of the 83% who saw the strategy as “stepping blocks” done together, one commented “Almost always you need to put those two together to make sure we’re doing things correctly and have a way to measure” (P02). Similarly, the “Develop and implement tools for quality monitoring” strategy was perceived as a stepwise process: “those are done sort of sequentially, but part of the same process…Because you can’t implement something you haven’t developed yet” (P10). Likewise, participants explained that certain multi-barreled strategies *should* be done together. For instance, one participant clarified (about the strategy “obtain and use feedback”), “You shouldn’t obtain feedback if you’re not going to use it for anything, but I think a lot of times we do. We ask for feedback and then we do nothing with it” (P10). Likewise with the strategy “Obtain and use patients/consumers and family feedback”, one participant explained, “somebody in this facility obtains feedback, but I don't know what they do with it” (P06).

#### Some strategies should be disaggregated into parts

In contrast, the five strategies that could be disaggregated into multiple parts were focused on resources and knowledge exchange. Participants noted that these compound strategies often were missing clarifying information, such as an intermediate step, details about who would do each part, or the intended outcomes. For example, one participant thought that “Capture and share local knowledge” may involve an intermediate step to “find out what your audience knows” (P12). In contrast to the obtaining and using feedback strategy, participant recommended "capture and share local knowledge" were actions meant to be split.

## Discussion

These cognitive interviews with clinicians identified how ERIC-based surveys can be made more acceptable and understandable for end-users. We identified strengths of the ERIC survey, including the comprehensiveness, unintended positive consequences, and ability to gather useful data. We also identified areas of confusion that can be easily addressed through wording and organization changes. Incorporating feedback such as adding project-tailored labeling and definitions may improve ease and usability of the survey, reduce confusion, and decrease participant burden. These pragmatic improvements to the ERIC survey could ultimately assist VA and other institutions in designing, evaluating, and replicating quality improvement efforts.

The ERIC survey has helped to advance data collection and the science of selecting implementation strategies. We previously demonstrated the face validity of the ERIC survey and identified strategies associated with better performance on EBPs over time [14]. For example, analyses showed that using more strategies was associated with more HCV treatment starts and yet some strategies were more impactful early in the initiative [6, 7]. Recent work has also reinforced the survey’s concurrent validity through interviews with respondents about their local activities [15]. These cognitive interviews demonstrated that there were unintended benefits of responding to the survey. Not only did certain uncommon but feasible strategies in the VA context prompt participant interest for QI planning, but the survey format assisted with within and cross year tracking efforts.

While there is no shortage of recent calls for clarity of strategies to improve precision implementation, complete characterization of strategies is possible only when there is a clear taxonomy. Therefore, consistent naming conventions, as pioneered by the ERIC project, are needed, as are discernable core strategy specifications. The ways in which individuals attach meaning to words is grounded in their experience, such that clinicians and implementation scientists interpret strategies differently. We generally found that clinicians were frustrated with implementation terminology, and certain potentially innocuous terms were shaped by clinical experience. This resulted in some terms being imbued with unanticipated meaning (“visit”) and others being rendered meaningless to clinicians (“facilitation” and “facilitator”). Likewise, we found that specific clusters of ERIC strategies were more confusing to clinicians than others leading to potential underreporting of strategy use. For example, the patient-facing strategies were easier for clinicians to understand (albeit were underspecified), while the differences in interactive assistance strategies were universally confusing to clinicians. More steps need to be taken to demystify implementation strategies to frontline providers and, conversely, to engage end-users in data collection strategy development.

Over the 8 years of fielding ERIC surveys, we have continued to grapple with the ongoing tension between making strategy assessments generic versus tailoring them to a specific context. ERIC was developed to create a generic taxonomy of implementation strategies to further cooperative learning across projects. Yet, strategies are expected to be tailored to the setting, making the adaptation of strategies both its own strategy and an important element of specification. Our findings further support the notion that generic strategy descriptions are poorly understood. There is thus a tension between maintaining universality versus providing specificity to make strategies more relevant and understandable. One solution may be including a project-tailored glossary with definitions that reflect the clinical innovation, setting, and actors [16].

Others have similarly recommended changes to the ERIC taxonomy. One such project, the School Implementation Strategies Translating ERIC Resources Project, made surface-level changes to 52 of the strategies, deeper changes to five, deleted six, and added seven new strategies [17]. Perry et al. refined definitions of 13 strategies and proposed three new strategies in the context of primary care cooperative: “assess and redesign workflow, create online learning communities, and engage community resources” [18]. We found additional overarching themes by talking with healthcare workers. There is a need for such efforts to learn from each other to advance the science of implementation.

In strategy assessment, there is a tension between decreasing the survey length and being comprehensive. We included all 73 strategies, in part, as a validity check and to also not omit potentially important but unanticipated strategies. However, these interviews highlighted ways that including uncommonly used strategies (in our case, financial strategies) may have inadvertently deterred survey participation. In contrast, deciding on strategy inclusion based on a priori perceptions of feasibility is likely inappropriate, given our findings that respondents’ perceptions of feasibility do not match those of researchers [5]. One potential solution may include presenting strategies that are perceived to be less feasible later in the survey. We have also changed the survey directions to ensure respondents know there is not an expectation they would have used all of the strategies. However, deciding which strategies to include in ERIC surveys requires more study.

One way to manage the large array of strategies is to be thoughtful about their presentation. Respondents were confused by the variable level of specification provided in the stems. ERIC includes multifaceted strategies that combine multiple discrete strategies and strategy bundles and ERIC strategies are variably specified (e.g., some include the actor and dose and others do not), which impacted interpretation. While there is a push for focusing on the mechanisms underlying the strategies [19–22], we found that clinicians wanted concrete, relatable activities to respond to. The same strategies can be used for different purposes, and different strategies may be targeting the same mechanism of behavior change. Future work should focus on organizing the strategies in ways that are understandable to providers and in ways that address both form and function. Likewise, strategy combinations and sequencing are important elements that are challenging to capture in simple surveys [23, 24]. Though we have addressed this (in part) through annual surveys across implementation efforts, this is not always feasible. Ultimately, strategies likely need to be disaggregated to core components and mechanisms as to enhance specificity.

### Strengths and limitations

These cognitive interviews with ERIC survey respondents provide novel insights into how these data should be collected. Participants were individuals who had completed at least three ERIC surveys, those with fewer or no experience with ERIC may have had even more difficulty understanding than presented here. Therefore, the changes that we make will need to be vetted with providers who are “survey naïve” and those in other disciplines, as we further adapt and refine the survey and associated methods. Selecting staff with more managerial and/or leadership positions may have yielded different results. Recall bias was cited as limitation to responding to annual surveys and was likewise a limitation here. Given this work was entirely in the VA, some findings may be less applicable to other settings. For example, financial strategies may be more applicable outside of the VA.

### Future work

Emerging and existing tools can help lay practitioners enter implementation science, report strategies, and enhance translation of strategy information across different groups [25–28]. A pragmatic implementation strategy reporting tool is in process now by Rudd and colleagues [29] and may aid in strategy use and specification among those with no specialized implementation science training. Similarly, Walsh-Bailey et al. have tested pragmatic strategy reporting tools with varying degrees of detail and found them to be largely acceptable, appropriate, and feasible [30]. We will also consider strategy de-implementation reporting in the future [31].

## Conclusion

This study identified ways in which ERIC strategy surveys can be improved for use in clinical settings. These findings contribute to the ongoing efforts to correct and improve the inventory of implementation strategies.

## Supplementary Information

**Additional file 1.**

## Data Availability

Data are available upon reasonable request from the corresponding author.

## Abbreviations

EBP: Evidence-based practice
ERIC: Expert Recommendations for Implementing Change
VA: Veterans Health Administration
HCV: Hepatitis C virus
QI: Quality improvement
RADaR: Rigorous and accelerated data reduction
